# Professional identification in the beginning of a teacher’s career: a longitudinal study on identity formation and the basic psychological need for autonomy in VET teacher training

**DOI:** 10.3389/fpsyg.2023.1196473

**Published:** 2023-08-03

**Authors:** Julia Katharina Weiß, Matthias Bottling, Tobias Kärner

**Affiliations:** Chair of Economic and Business Education (560A), University of Hohenheim, Stuttgart, Germany

**Keywords:** vocational education and training, teacher identity development, professional identification, autonomy support and thwarting, longitudinal study, cross-lagged panel analyses, intention to stay

## Abstract

**Introduction:**

This study examines the extent to which VET trainee teachers’ identification with their profession is related to their basic psychological need for autonomy and whether this is reflected in their intention to stay in the field. Trainee’s subjective experience of their professional identity interacts with different conditions of the training environment, whereby we focus on perceived autonomy support and autonomy thwarting behavior of seminar teachers.

**Methods:**

On the basis of a longitudinal design with a total of 79 trainee teachers in Germany and four survey time points during teacher training, corresponding developmental processes were traced over a total period of 1 year. Cross-lagged panel analyses allow us to draw conclusions about the extent to which professional identification of trainees interacts with autonomy-support or autonomy-thwarting conditions originating from seminar teachers and to what extent the aforementioned factors in turn affect intention to stay.

**Results:**

Cross-lagged panel analyses show that professional identification after 6 months in teacher training significantly predicts the intention to stay in the teaching profession half a year later. Significant cross paths each describe positive effects between professional identification and autonomy support and negative effects between professional identification and autonomy thwarting.

**Discussion:**

Particularly against the background of the shortage of teachers in Germany and other countries, the promotion of professional identification processes in the sense of a teacher identity can be assessed as crucial. In this respect, an autonomy-supporting environment, e.g., created by seminar teachers, can already contribute to that during teacher training.

## 1. Introduction

The transition into professional life induces a further development of the personal identity through the professional social context (Heinrichs et al., 2022). Especially the phase of teacher training is probably the most formative time in a teacher’s career. Besides numerous, unfamiliar tasks and a new work environment, trainee teachers face the challenge of developing their own professional teacher identity. The influence of various factors on the construction and evolution of teachers’ professional identity is evident across different international contexts during the initial teacher training period (Gracia et al., 2022). The concept of teacher identity, which involves beliefs of beginning teachers about occupational group specific actions and norms as it relates to their own identity as a teacher, is of utmost importance to teacher education (Bullough, 1997). The establishment of a professional identity can be regarded as a crucial component and consequence of vocational education programs, with the assumption that it influences trainees’ acquisition of knowledge and their performance (Klotz et al., 2014). In addition, the development of a “teacher personality” is demanded as a goal of professionalization in the course of teacher training, since this identity formation is also critical to students’ learning success (Rothland, 2021). Not only the mere subject-specific expertise of teachers has an influence on successful student learning, but also teacher personality attributes can be regarded as crucial (Klassen and Tze, 2014; Loebell and Martzog, 2017). Meyer (2020) suggests that professional identity in particular should be considered to assess successful professionalization, i.e., in the sense of a competence-theoretical determination approach a definition of competence areas and knowledge dimensions, which are important or necessary for the accomplishment of the tasks within the teaching profession (Terhart, 2011). For professionalization, continuous reflection on the professional situation by the trainees themselves is of great importance (Cramer et al., 2023). Also, seminar teachers^1^ contribute substantially to this professionalization, as they constantly advise, foster, and evaluate their trainees (Kärner et al., 2022).

A prerequisite for trainee teachers to be able to identify with their newly joined profession is that they are granted sufficient freedom in their professional and personal (further) development during their training (Orr, 2012; Kaplan et al., 2021). Autonomy-supportive procedures can facilitate an environment wherein trainees engage in self-reflection and embark upon an exploratory journey, ultimately contributing to the construction and expression of their individual identities (Kaplan et al., 2021). Against this background, it seems to be reasonable to examine the autonomy experience of trainee teachers in more detail. Also, in educational science discussions, autonomy is emphasized as one of the constitutive characteristics of professionalism (Meyer, 2020). The perception of autonomy among teachers is what determines their professional identity and is also a crucial factor in comprehending this identity (Siraj-Blatchford, 1993). There are various studies demonstrating that autonomy-supportive teacher behavior has a positive impact on, for example, achievement or engagement of learners (e.g., Reeve et al., 2004; Hornstra et al., 2021). In the context of teacher training, trainee teachers also assume the role of learners, and their relationship with the seminar teacher bears resemblance to the teacher-student dynamic. Since seminar teachers are responsible for training practice and content as well as assessment, they play a central role in granting scope for action on the one hand and introducing binding, sometimes even restrictive guidelines on the other. Moreover, having a strong professional identification as well as teacher autonomy in terms of scope for decision-making and action can be beneficial in reducing the likelihood of dropping out and increasing the desire to stay committed to the professional field (e.g., Weiss, 1999; Ingersoll, 2001; van Dick and Wagner, 2002; Hong, 2010; Avanzi et al., 2014; Grant et al., 2020; Fradkin-Hayslip, 2021). The *intention to stay* in the profession can be used to measure the desire to remain in the field and can be viewed as an indication that individuals are at least not considering quitting or changing professions, which thus differs from the concept of dropout intention. The latter can be employed as a predictor of actual dropout (see Krötz and Deutscher, 2022).

So far, studies on identity formation, both in general (Heinrichs et al., 2022) and in the context of teacher training, have mainly been cross-sectional in nature. However, the period of teacher training is characterized by a continuous process of professional development, which is shaped by ongoing self-reflection as well as assessments from others, such as seminar teachers. This dynamic process has the potential to significantly influence the trainee’s professional identification processes.

Against this background, in our study, we aim to investigate the extent to which VET (vocational education and training) trainee teachers’ identification with their profession is related to their basic psychological need for autonomy and whether this is reflected in their intention stay in the professional field. Therefore, the study objective is to empirically examine the plausibility of different possible effects between professional identification and either support or thwarting of autonomy, as per their theoretical justification. On the basis of a longitudinal design with a total of 79 trainee teachers in Germany and four survey time points during teacher training, we traced corresponding developmental identity processes as well as the perception of an autonomy supportive or an autonomy thwarting environment. Cross-lagged panel analyses allow the investigation of time-dynamic interactions of the considered constructs. In order to pursue our research aim, theoretical approaches and empirical findings on professional identification as well as autonomy support and autonomy thwarting in basic psychological need theory are outlined (Sections 2.1 and 2.2). Besides their general theoretical foundation, the reference to teacher training is established and the link to intention to stay is drawn. Thereafter, the complex interplay between these two conceptual foci, which constitutes the substantive primary concern of this article, is discussed theoretically by drawing on relevant theories (Section 2.3). Ultimately, the research aim and hypotheses are derived (Section 3). The empirical results (Section 5) form the basis for the final discussion of the findings, and the study concludes by highlighting implications for further research and practical applications for VET teacher training (Section 6).

## 2. Theoretical framework

### 2.1. Professional identification of trainee teachers

When individuals identify with their profession, they perceive themselves as members of their occupational group as well as define themselves through their work and the associated typical characteristics of this profession (Mael and Ashforth, 1992; van Dick and Wagner, 2002). According to Kirchknopf and Kögler (2020), professional identity is conceptualized as a part of social identity and thus of identification with the profession. Professional identification is relevant for identity development, since identification is about integrating this group membership into one’s self-concept (Haslam et al., 2000). Identity development is a lifelong process that manifests itself in various areas of life, including work life. Thus, professional identity is a facet of overall identity that pertains to the domain of occupation and career (Erikson, 1959; Marcia, 1980). The focus of this article lies primarily on the (social) identity or identification with a group or entity, which in this case concerns the professional group of teachers or the teaching profession in general. Both terms, identity and identification, are closely related, as they each influence the way individuals perceive themselves and their relationship with others. The starting point for the formation of an individual’s identity constructions lies in the *self*. The development of the self is an ongoing process that ultimately enables the individual to understand the dynamics of the mutability of the self and identify a personal core that is equally characterized by continuity as well as stability and constitutes an *identity* (Greve, 2007; Heinrichs et al., 2022). *Identification* refers to the process by which a person identifies with a particular role (e.g., being a teacher), group (e.g., professional group of teachers), organization (e.g., a specific school), or other entity (e.g., teaching profession) and thereby partially shares his or her identity with others. This can involve a cognitive, psychological, or emotional connection between the individual and the entity in question. In other words, the concept of identity primarily pertains to an individual’s internal sense of self, while identification is a more outwardly-focused construct (Miscenko and Day, 2016).

In the context of describing and modelling professional identity formation and development, the *Social Identity Approach* serves as a central theoretical reference, encompassing *Social Identity Theory* (SIT) and *Self-Categorization Theory* (SCT). The main assumption of both theories is that an identity consists not only of an individual personality, but also of the membership in social groups (Schuh et al., 2021). The perception of one’s self is decisively influenced by this affiliation and impacts behavior, attitudes, and the individual self-concept. According to the SIT, people strive for a positive self-evaluation, which is in part determined by belonging to a social (professional) context. The basis of this self-assessment is social identification, which can be positive or negative depending on how the groups that contribute to a person’s social identification are evaluated (Tajfel and Turner, 1979, 1986). Belonging to a group can thus elicit positive feelings such as pride, but also negative feelings such as frustration or hostility toward other groups (Ashforth and Mael, 1989). The SCT represents a further development of the SIT and emphasizes that people not only derive their identity from belonging to certain social groups, but that they can also change their self-categorization depending on the context (Turner et al., 1987). According to this theory, people see themselves as members of certain categories depending on which distinctions are most relevant in a given situation (van Dick et al., 2004). This means that people define their identity based on the characteristics they share with other members of a category, rather than just on their individual characteristics. In summary, SIT focuses on the importance of belonging to a social group in identity formation, while SCT addresses the significance of the characteristics and distinctions that constitute the membership in a group. Overall, the Social Identity Approach can help us understand the relevance of group identities in the workplace and how these identities can influence employee cognition, behavior, and emotion (van Dick et al., 2004).

In vocational and business education, professional identity constructs are closely related to the idea of vocation (Heinrichs et al., 2022). Beck (2018) suggests an approach that employs the term “vocation” as a framework for task-related metacognitions, encompassing aspects of an individual’s task-related self-conception (e.g., *meaning cognitions*, i.e., the basis of one’s own motivation for work, *relevance cognitions*, such as social significance or *competence cognitions* in the sense of qualifications and willingness) that have been explored to date, while also offering key intervention opportunities for vocational education and training programs aimed at facilitating successful professional integration. According to Beck (2018), the concept of vocation in its six-dimensional manifestation can be seen as a partial differentiation of what is called “professional identity” in the literature. The occupation pursued is assessed and reflected upon by the individual (Beck, 2018). Vocation can, to a certain extent, have an identity-forming effect, and thus the vocational awareness of trainees in the form of task-related metacognitions is related to professional identity (Beck, 1997; Kirchknopf and Kögler, 2020).

Various studies on teachers have found that professional identification is associated with many positive effects, such as perceptions of greater social support, higher job satisfaction or fewer symptoms of stress (van Dick and Wagner, 2002; Bizumic et al., 2009; Avanzi et al., 2015). Moreover, professional identification and intentions to stay are closely related. Strong professional identification, i.e., a strong sense of belonging and commitment to the occupation, can help reduce intentions to dropout and increase intentions to stay. A lack of professional identification, by contrast, can lead to dissatisfaction and a greater desire to change occupations. Therefore, fostering strong professional identification among beginning teachers can be an important factor in minimizing intentions to dropout and promoting intentions to stay (e.g., van Dick and Wagner, 2002; Hong, 2010; Avanzi et al., 2014).

### 2.2. Basic psychological need for autonomy

#### 2.2.1. Autonomy support and autonomy thwarting in basic psychological need theory

Autonomy encompasses the sense of independence and personal agency in making choices as well as having control over one’s own actions and decisions (Ryan and Deci, 2006). As one of the six mini-theories within *Self-Determination Theory* (SDT), *Basic Psychological Need Theory* (BPNT) plays a crucial role in explaining “how need support promotes and need thwarting undermines healthy functioning at all levels of human development and across cultural backdrops and settings” (Ryan and Deci, 2017, p. 21). BPNT considers three basic psychological needs, namely autonomy, competence, and relatedness, which are essential for the development of self (Deci et al., 2013). While the focus within BPNT was initially solely based on need satisfaction, both methodologically and conceptually it was increasingly also put emphasis on need frustration, so that a two-dimensional approach emerged (Vansteenkiste et al., 2020). The relationship between need satisfaction and frustration is asymmetrical, where the lack of need satisfaction does not necessarily mean the presence of need frustration, but the presence of need frustration indicates the absence of need satisfaction (Vansteenkiste and Ryan, 2013; Vansteenkiste et al., 2020).

Similar to the distinction made between experiences of need satisfaction and need frustration, social or work-related environments can be distinguished as being either *need supportive* or *need thwarting*. This means that agents of professional socialization can actively promote, remain indifferent to, or work against the individual’s satisfaction of needs (Vansteenkiste and Ryan, 2013). Therefore, the specific context, which corresponds in our study to the teacher training program, plays a decisive role as it can (pro-)actively support or thwart basic psychological needs (for example, one could imagine a situation in which a seminar teacher gives his/her trainees little or no scope for individual preferences in preparing lessons, thus undermining their autonomy). The SDT model on need satisfaction and need frustration illustrates the link between need support and need satisfaction as well as need thwarting and need frustration, respectively: while need-supportive environments lead to the experience of need satisfaction and, in turn, to personal growth and well-being, need thwarting environments lead to the experience of need frustration and thus, to developmental dysfunction and ill-being (Vansteenkiste and Ryan, 2013).

According to Vansteenkiste et al. (2020), autonomy support has historically been the focus of attention and research interest, primarily due to two reasons. Firstly, the need for autonomy is a fundamental aspect of SDT and it is also a subject of controversy. Secondly, socializing agents who are supportive of autonomy tend to be responsive to the needs of competence and relatedness. The importance of autonomy-supportive and autonomy-thwarting behavior in the teaching-learning context was already highlighted by numerous studies. For example, Jang et al. (2016) found that students who were taught in their preferred way of teaching perceived their teacher as being more autonomy supportive. Moreover, these individuals reported significantly higher levels of autonomy need satisfaction, engagement, and conceptual learning. Vermote et al. (2020) found that teachers in higher education who are autonomously motivated themselves tend to adopt a need-supportive teaching style. In contrast, teachers who exhibit controlled motivation tend to adopt a need-thwarting teaching style. And finally, Assor et al. (2020) suggest several autonomy relevant practices, such as facilitating decision-making, that educators could implement in order to foster identity development.

#### 2.2.2. Autonomy support and autonomy thwarting in teacher training

Teacher training in Germany is generally divided into two parts. The first phase takes place at a university or a college of education, while the second, practical phase is referred to as preparatory service or teacher training. This second phase of teacher education, which lasts 18 months in the federal state of Baden-Württemberg (Germany), takes place at two learning sites, namely the training school and the college of didactics and teacher education. In the latter, *seminar teachers* are in charge of teaching didactics and teaching skills in specific subjects as part of their training as well as evaluating their trainees’ performance. Therefore, they fulfill the dual function of an advisor and an assessor, which in turn poses the risk of inter-role conflicts (Kärner et al., 2021a, 2022). In particular, in the first half of the training period, the role of the advising teacher is predominant among seminar teachers, while it then transitions to a phase in which many grade evaluations by seminar teachers are due and the performance of trainees is closely monitored, which represents a shift in the role of seminar teachers from advisors to assessors. This dual role of seminar teachers implies a *dependency* of the trainees on them, which in turn can partially limit their experience of autonomy. Therefore, trainee teachers may experience ambivalence when interacting with the same person, perceiving both heteronomy as well as a source of support (Prengel, 2019; Kärner et al., 2021b, 2022). Seminar teachers form an essential part of the trainees’ learning and working environment and, therefore, might also contribute to the development of the trainee’s sense of self and professional identity. The self is fundamentally relational in nature, i.e., it is intimately connected with *significant others* (Andersen et al., 2002). Therefore, essential elements of the self are perceived and experienced in relation to these significant others, which, in addition to the usually meant spouse or partner, also applies to peers and others who have a profound influence on the individual (such as seminar teachers within the context of teacher training) (Kärner et al., 2021b). Just as the significant other is of substantial importance to the development of the self, this reference person could be equally important to the development of professional identity in the professional context.

Numerous studies show that autonomy-supportive teacher behavior has a positive impact on multiple learner-related variables, such as motivation, achievement, or engagement (e.g., Reeve et al., 2004; Hornstra et al., 2021). Within the realm of teacher training, trainee teachers not only fulfill the role of aspiring educators but also adopt the position of learners. Consequently, their interaction with the seminar teacher exhibits great similarities to the teacher-student dynamic. Therefore, it seems plausible that autonomy-supportive behavior of seminar teachers can also affect professional identification of trainees over time (Kirchknopf and Kögler, 2022) or that a firmly established identity is in fact a prerequisite for general acceptance in the school context (e.g., by seminar teachers), ultimately enabling trainees to autonomously follow their own path or strive for their own goals (Derakhshan et al., 2020). In another VET setting, Klotz et al. (2014) demonstrated that industrial apprentices who were granted the opportunity to participate in all operational procedures not only more easily cultivated their professional identity but also exerted greater workplace effort, highlighting the significance of an autonomy-supportive environment. It has already been shown that trainee teachers are caught between the conflicting priorities of desired alignment and fulfillment of expectations on the one hand and seeking autonomy on the other, and that in some cases they perceive excessive power asymmetries towards their seminar teachers, which is then associated with lower levels of satisfaction and a higher experience of stress (Kärner et al., 2022).

In addition, experiencing autonomy can increase trainee teachers’ engagement and satisfaction, which in turn can make them more likely to want to stay in their profession. When teachers feel they have more control and influence over their work, they are able to shape their own way of working and make better use of their skills and abilities. This can help them feel that their work is meaningful and fulfilling or that they can better identify with their profession, which is likely to increase retention (e.g., Weiss, 1999; Ingersoll, 2001; Grant et al., 2020; Fradkin-Hayslip, 2021). Weiss (1999), for instance, found that teachers in their first year who perceive a sense of autonomy are those who feel empowered to influence content, teaching techniques, and discipline methods. Such teachers are more likely to choose to continue a career in teaching and plan to remain in the profession.

### 2.3. Interrelationship between professional identification and autonomy

From the perspective of developmental psychology, professional identity varies and develops over the lifespan (e.g., Watson, 2006; Hong, 2010). Due to the mutual influence of changing environmental conditions such as the inhibition or support of autonomy and individual self-reflective processes, the subjective experience of professional identity can change (Luyckx et al., 2009; Heinrichs et al., 2022). Primarily, identity-forming conscious content determinations are those that have been chosen voluntarily and autonomously by a person or to which he or she at least agrees in deepest conviction (Warwas, 2012). According to SDT, the satisfaction of basic psychological needs, as the result of a need-supportive environment, is particularly important for the internalization or personal acceptance of the chosen identity, so that the adopted identity can emerge from the sense of self (Skhirtladze et al., 2019). The fulfillment of these basic needs consequently affects identity commitment and is associated with higher professional identification (Luyckx et al., 2009).

However, it is precisely the connection between the *need for autonomy* and the feeling of identification that entails a complex interplay since they can both be interdependent but also be mutually exclusive. Gruen (1968) argues that autonomy, or the ability to act independently and make one’s own decisions, is essential for a healthy sense of self. However, he also acknowledges the importance of identification, or the sense of belonging and connection to others. The *paradox*, as the author sees it, is that autonomy and identification can contradict each other. On the one hand, identification can lead to a loss of autonomy as individuals conform to group norms and values. Indeed, having a high level of identification can limit a person’s autonomy if they are required to adhere to certain rules in order to remain a part of a group (Rudolf, 2017). On the other hand, autonomy can result in a sense of isolation and disconnection from others (Gruen, 1968). This contradiction, which is actually traced back to the parent–child relationship, nevertheless appears to be fundamentally applicable to the context of teacher training. In the case of trainee teachers, the professional identification process (among other things, putting oneself in the position of being part of the professional group of teachers and educators as well as internalizing corresponding role conceptions and images, etc.) can encounter the individual striving for independence or the desire to become and be an independent part of precisely this context, i.e., to want to make oneself independent in a situation of dependence as per educational status. Autonomy and identification are thus interrelated, but they influence each other in different ways and can also be in tension (Gruen, 1968; Rudolf, 2017).

Another facet of the paradoxical relationship between identification and autonomy is described by Helsper (2016) in the context of a so called “autonomy-antinomy” in relation to professional action. If professionals (e.g., seminar teachers) act in a supportive and facilitating way to enable autonomy and therefore potentially enhance identification processes, this can paradoxically lead to strengthening dependency, dissolving already existing autonomy, and thus generating heteronomy. Besides the social context in which “professionals” influence their trainees, the *intrapsychic perspective* must also be considered. With reference to psychodynamic approaches, a basic inner psychological conflict in the area of tension between *individuation* and *dependency* is assumed, or, with reference to the work context, in the conflicting relationship between *attachment* and *autonomy*. According to the psychodynamic theory, a person in the “passive” (avoidant) mode is existentially dependent on emotional attachments, whereas a person in the “active” (assertive) mode is more likely to strive to maintain autonomy in any situation in life and to avoid close emotional relationships and thereby possibly preventing (too strong) identification with others. A person who is free of this conflict can enter into emotionally significant relationships and yet at the same time experience himself/herself as autonomous and self-reliant (Benecke and Möller, 2013, 2019; Kotte et al., 2019). This is especially relevant if one assumes that social relationships play at least a partial role in identifying with the profession. The latter, so-called “integrated” mode may mean for trainee teachers that they identify with the teaching profession and the incorporated roles they take on while still perceiving themselves as self-determined.

In summary, at least two possible mechanisms are conceivable, which are suitable to describe interrelationships between professional identification of trainee teachers and the support or thwarting of the basic need for autonomy provided by the seminar teacher:

- On the one hand, it can be assumed that the support or thwarting of the basic need for autonomy has an effect on professional identification. The satisfaction of autonomy enables individuals to behave in a way that allows them to pursue their own desires and needs. Consequently, autonomy-supportive environments allow trainee teachers to experience various possibilities of identifying with the profession in order to eventually find and integrate their personal teacher identity into their self (e.g., Luyckx et al., 2009; Helsper, 2016; Assor et al., 2020). Theory also shows that a strong sense of autonomy may result in less identification (Gruen, 1968), as a high degree of autonomy has the potential to engender a sense of isolation and detachment from one’s professional social environment.
- On the other hand, it can be assumed that the degree of professional identification has an effect on the perception of the support or thwarting of the basic need of autonomy. A high level of identification may limit a person’s autonomy if he or she must follow certain rules and norms in order to remain part of a (professional) group (Gruen, 1968; Helsper, 2016). Conversely, strong identification can also provide a sense of belonging and social support, which in turn can promote the perception of autonomy support (e.g., Schuitema et al., 2016; Kim et al., 2019).

## 3. Research aim and hypotheses

Up to now, there have been mainly cross-sectional studies about identity in general (Heinrichs et al., 2022) and in particular in regard to teacher training. However, especially during teacher training, the trainee experiences a constant professional development, which is shaped by continuous self-assessments and assessments by others (e.g., seminar teachers), potentially influencing professional identification processes. With this in mind, in our study we consider professional identification from a developmental perspective as well as temporal reciprocal dependencies with the factors described above. The objective is to empirically examine the plausibility of the different possible effects between professional identification and either support or thwarting of autonomy, as per their theoretical justification. Besides the link to intention to stay is drawn.

First, we consider those hypotheses that refer to the time-based relationship between perceived autonomy support or thwarting and professional identification, testing the first two hypotheses against each other (see Section 2.3):

*H1:* A *higher* level of perceived autonomy support through seminar teachers (T*_n_*)^2^ leads to *higher* professional identification among trainees (T_*n*+1_).

*H2:* A *higher* level of perceived autonomy support through seminar teachers (T*_n_*) leads to *lower* professional identification among trainees (T_*n*+1_).

*H3:* A *higher* level of perceived autonomy thwarting through seminar teachers (T*_n_*) leads to *lower* professional identification among trainees (T_*n*+1_).

Second, regarding the time-based relationship between professional identification and perceived autonomy support or thwarting, two competing hypotheses need to be tested against each other:

*H4:* Assumption of a *coherent* effect: A *higher* professional identification among trainees (T*_n_*) leads to a *higher* perception of autonomy support or a *lower* perception of autonomy thwarting through seminar teachers (T_*n*+1_).

*H5:* Assumption of a *contradictory* effect: A *higher* professional identification among trainees (T*_n_*) leads to a *lower* perception of autonomy support or a *higher* perception of autonomy thwarting through seminar teachers (T_*n*+1_).

Third, the final hypotheses refer to associations between professional identification as well as perceived autonomy support or thwarting and the intention to stay in the profession (see Sections 2.1 and 2.2):

*H6: Higher* professional identification during teacher training (T*_n_*) leads to a *higher* intention to stay in the profession after one year (T_4_).

*H7: Higher* perceived autonomy support or *lower* perceived autonomy thwarting through seminar teachers during teacher training (T*_n_*) leads to a higher intention to stay in the profession after one year (T_4_).

## 4. Method

### 4.1. Data collection and sample

**Figure 1:**
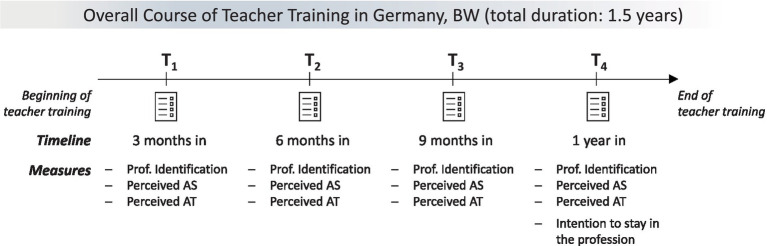
Study design and measures. BW, federal state of Baden-Württemberg; Prof. Identification, professional identification; AS, autonomy support (provided by seminar teachers); AT, autonomy thwarting (through seminar teachers).

On the basis of a longitudinal design^3^ with a total of 79 trainee teachers in Germany and four survey time points during teacher training (3 months in, 6 months in, 9 months in, 1 year in, see Figure 1 for the timeline and measures used for each time point), corresponding developmental identity processes were traced. In total, this amounts to 234 single measurements.

The sequence of the training was identical for all trainee teachers with regard to the general organizational structure. A corresponding standardization of the general temporal and organizational procedures ensures transparency with regard to unobserved events between the individual measurement points on the one hand and is important with regard to the interpretative robustness of the effects found on the other.

The four time periods included can be characterized in terms of their general organizational conditions and training content as follows:

- *Measurement at T_1_ (beginning of teacher training up to 3 months):* “Onboarding,” general information about the training, educational theory lessons at the central training institution (seminar) in a four-week block format, followed by orientation at the training school; implementation of the first teaching lessons mainly in supervision of mentors and staff.
- *Measurement at T_2_ (3 months in to 6 months in):* Training alternately at the central training institution (seminar) and at the training schools, preparation and delivery of first independent lessons under supervision of the seminar teacher.
- *Measurement at T_3_ (6 months in to 9 months in):* Training alternately at the central training institution and at the training schools, independent teaching and responsibility for own classes.
- *Measurement at T_4_ (9 months in to 12 months in):* Preparation for the examination period and assessment phase, planning, implementation and evaluation of an individual teaching unit, which must be written down and is subsequently graded.

The sample is a full census of one of a total of four training institutions for teachers at vocational schools in the federal state of Baden-Württemberg (BW), Germany. The study was approved by the responsible governmental authority and all participants gave their consent concerning the participation in the study and with regard to processing their data. The sample is representative of the training cohort with the same training start date in the named federal state, as our sample consists of 54 females (62.79%), which reflects the distribution of female trainee teachers in BW (60.05%). A chi-squared analysis confirmed that the proportion of males and females does not differ significantly from the gender distribution of the other VET training institutions in BW [χ^2^(1, *n* = 388) = 0.346, *p* = 0.557]. Further, the average age of the sample (29.63 years) corresponds to the average age of the population (29.70 years). When comparing our sample with the other VET training institutions a *t*-test revealed that there is no significant average age difference [*t*(380) = 0.140, *p* = 0.889].

### 4.2. Measures

Drawing upon the existing literature, we utilized different measurement instruments that corresponded to distinct theoretical concepts relevant to our study. The following instruments were used to measure the variables of interest:

- *Professional identification* scale was adapted from van Dick and Wagner (2002) as well as van Dick et al. (2004) and measured by eight items (range of Cronbachs α [0.83; 0.89], e.g., “For the past 3 months of my traineeship, I considered myself a member of my professional group.”) on a six-point Likert-type scale ranging from (1) *does not apply* to (6) *applies completely*.
- *Perceived autonomy support from seminar teachers* was measured by three items via an adapted scale from Gilbert et al. (2021) (range of Cronbachs α [0.75; 0.84], e.g., “In the past 3 months of my traineeship, my seminar teachers encouraged me to follow my own path.”) on a six-point Likert-type scale ranging from (1) *do not agree* to (6) *totally agree*.
- *Perceived autonomy thwarting from seminar teachers* was measured by three items via an adapted scale from Gilbert et al. (2021) (range of Cronbachs α [0.84; 0.89], e.g., “In the past 3 months of my traineeship, my seminar teachers pressured me to do things their way.”) on a six-point Likert-type scale ranging from (1) *do not agree* to (6) *totally agree*.
- *Intention to stay in the teaching profession* was measured by a self-formulated single item (“At the moment I plan to stay in the teaching profession after finishing teacher training”) on a five-point Likert-type scale ranging from (1) *does not apply* to (5) *applies completely*.

### 4.3. Data analysis

Besides descriptive statistics, Pearson product–moment correlations were calculated using SPSS 27 (IBM, Chicago, United States). Cross-lagged panel analyses were conducted using Mplus 8 (Muthén and Muthén, 1998–2017). Regarding the handling of missing values, the full information maximum likelihood method (FIML) was used, which is implemented in Mplus as a standard routine. Enders and Bandalos (2001) found that in structural equation models, FIML estimation outperforms other methods (i.e., listwise deletion, pairwise deletion, and response pattern imputation) in various outcome measures, such as parameter estimate bias or model goodness of fit conditions. FIML was unbiased and efficient under ignorable missing data conditions and had the lowest proportion of convergence failures and near-optimal Type 1 error rates. It is important to mention that individuals who stopped participating in our survey continued to actively attend teacher training and have not withdrawn from the program; they have merely stopped their participation in our specific study, resulting in what is commonly referred to as panel attrition, making the FIML method an appropriate means of dealing with our missing values.

Time-dynamic analyses allow us to draw conclusions about the extent to which professional identification of trainee teachers for vocational schools and autonomy-promoting or autonomy-inhibiting factors are related to each other over time, and to what extent the aforementioned factors in turn affect intention to stay. In general, cross-lagged panel models prove to be particularly valuable in tracking the relationships between variables over time, or in the context of developmental science. Furthermore, corresponding models are suitable for the analysis of causal relationships if these cannot be verified on the basis of experimental settings. Corresponding statistical approaches are based on the idea of so-called Granger causality (Granger, 1980). This assumes that if a predictor is clearly responsible for the time-based prediction of a variable, this circumstance serves as preliminary evidence for causality (Zyphur et al., 2020). In detail, the autoregressive effects in the cross-lagged panel model refer to the consistency of the constructs over time, specifically reflecting the consistency of individual differences from one instance to the next. Instead, cross-lagged effects represent the influence of a construct on another measured at a subsequent time point, while also accounting for the previous level of the predicted construct. By controlling for prior levels of the outcome construct, one can eliminate the chance that a cross-lagged effect at time *t* + 1 is solely a result of two variables being correlated at time *t* (Selig and Little, 2012).

## 5. Findings

### 5.1. Descriptive data and Pearson correlations

Table 1 contains descriptive data (means and standard deviations) as well as Pearson correlation coefficients. With respect to descriptive scale values, we find that the mean values for professional identification, autonomy support and intention to stay are above, mean values for autonomy thwarting below average across the four measurement time points. Regarding standard deviations of the individual scales, it is noticeable that on the one hand they tend to increase across the three constructs, starting with professional identification (0.750–0.834), via autonomy support (1.071–1.404) to autonomy thwarting (1.351–1.805), which indicates, among other things, considerable variations especially in perceived autonomy thwarting through seminar teachers. On the other hand, standard deviations also increase within the individual constructs across the four measurement time points, i.e., the individual perceptions scatter more according to the progress of teacher training.

**Table 1:** Descriptive data and Pearson correlations.

	Variables	*M*	*SD*	(1)	(2)	(3)	(4)	(5)	(6)	(7)	(8)	(9)	(10)	(11)	(12)
(1)	Professional identification (T_1_)	4.778	0.750												
(2)	Professional identification (T_2_)	4.738	0.791	**0.710**											
(3)	Professional identification (T_3_)	4.713	0.723	**0.537**	**0.679**										
(4)	Professional identification (T_4_)	4.630	0.834	**0.501**	**0.631**	**0.775**									
(5)	Autonomy support (T_1_)	4.258	1.071	**0.309**	**0.288**	**0.329**	**0.326**								
(6)	Autonomy support (T_2_)	4.272	1.109	**0.414**	**0.459**	**0.331**	0.256	**0.519**							
(7)	Autonomy support (T_3_)	4.294	1.245	**0.476**	**0.422**	**0.323**	**0.419**	**0.565**	**0.507**						
(8)	Autonomy support (T_4_)	4.068	1.404	0.140	0.102	0.115	0.092	0.085	0.157	**0.478**					
(9)	Autonomy thwarting (T_1_)	2.724	1.351	**−0.283**	**−0.275**	−0.221	−0.180	**−0.815**	**−0.415**	**−0.422**	−0.063				
(10)	Autonomy thwarting (T_2_)	2.588	1.364	**−0.340**	**−0.254**	−0.166	−0.231	**−0.447**	**−0.608**	**−0.484**	−0.278	**0.491**			
(11)	Autonomy thwarting (T_3_)	2.585	1.440	**−0.400**	**−0.386**	**−0.301**	**−0.321**	**−0.313**	**−0.575**	**−0.458**	−0.268	**0.442**	**0.719**		
(12)	Autonomy thwarting (T_4_)	2.252	1.805	−0.125	**−0.370**	−0.127	0.006	**−0.393**	−0.253	**−0.382**	−0.169	**0.508**	**0.527**	**0.497**	
(13)	Intention to stay in the profession (T_4_)	4.400	0.857	**0.411**	**0.610**	**0.422**	**0.707**	0.283	0.227	0.172	0.112	**−0.287**	−0.283	**−0.316**	−0.028

Coefficients marked in bold are significant at least at the ≤ 0.05 level; 44 ≤ *n* ≤ 72; Professional identification: six-point Likert-type scale (1 = does not apply to 6 = applies completely); Autonomy support and autonomy thwarting: six-point Likert-type scale (1 = do not agree to 6 = totally agree); Intention to stay in the profession: five-point Likert-type scale (1 = does not apply to 5 = applies completely).

Regarding the correlations, as suspected, the scales for *professional identification* are strongly^4^ and significantly positively correlated with each other at all four measurement time points (0.501 ≤ *r* ≤ 0.775). One would also expect this pattern for the scales of *autonomy support* and *autonomy thwarting*. While the scales of autonomy thwarting also show moderate to strong significant positive correlations with each other across the four measurement time points (0.442 ≤ *r* ≤ 0.719), the scale of autonomy support at time point T_4_ is not associated with the scale of autonomy support at time points T_1_ and T_2_. The other measurement time points regarding autonomy support are significantly positively correlated with moderate to strong effect sizes (0.478 ≤ *r* ≤ 0.565).

The scale measuring perceived *autonomy support* provided by seminar teachers at time point T_1_ and T_3_ correlates moderately and significantly positively with the scale for *professional identification* at all four measurement points (0.288 ≤ *r* ≤ 0.476). Perceived autonomy support at time T_2_ correlates moderately and significantly positively with the scale for professional identification at measurement time points T_1_ to T_3_ (0.331 ≤ *r* ≤ 0.459). However, perceived autonomy support at time point T_4_ does not correlate with the scales for professional identification at all time points.

The scale measuring perceived *autonomy thwarting* through seminar teachers at time point T_1_ and T_2_ correlates moderately and significantly negatively with the scale for *professional identification* at measurement time points T_1_ and T_2_ (−0.340 ≤ *r* ≤ −0.254). Perceived autonomy thwarting at time T_3_ correlates moderately and significantly negatively with the scale for professional identification at all four time points (−0.400 ≤ *r* ≤ −0.301), whereas the scale for autonomy thwarting at time point T_4_ only correlates with the scale for professional identification at time point T_2_ (*r* = −0.370).

Finally, the *intention to stay* in the teaching profession measured at time point T_4_ is moderately to strongly and significantly positively correlated with professional identification at all four measurement time points (0.411 ≤ *r* ≤ 0.707). While intention to stay at time point T_4_ and autonomy support are not associated with each other for none of the four time points, the intention to stay in the teaching profession is moderately and significantly negatively correlated with autonomy thwarting at time point T_1_ (*r* = −0.287) and T_3_ (*r* = −0.316).

### 5.2. Cross-lagged panel models

**Figure 2:**
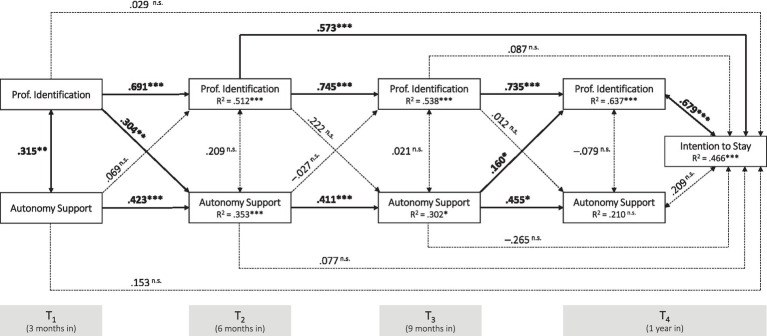
Cross-lagged panel model of professional identification and autonomy support at four time points and intention to stay at T_4_ (Model 1). ***p ≤ 0.001, **p ≤ 0.01, *p ≤ 0.05.

To track the interrelationships between professional identification and autonomy support or thwarting, respectively, across the four measurement time points, cross-lagged panel models were conducted. Figure 2 illustrates the relationship between the two variables *professional identification* and *autonomy support* across the different measurement time points, as well as their relationship to the variable *intention to stay* at time T_4_ (*Model 1*). Model fit indices indicate that the model fits the data well: *χ^2^* = 12.54, *df* = 12, *p* > 0.05 (with H_0_ = specified model, which therefore cannot be rejected); CFI = 0.997; TLI = 0.992; RMSEA = 0.024, RMSEA 90% CI: [0.00, 0.12]; SRMR = 0.053. Adequate proportions of variances in the dependent variables were explained by the independent variables in the model, which were larger for professional identification (with *R^2^* = 0.512 at T_2_, *R^2^* = 0.538 at T_3_, and *R^2^* = 0.637 at T_4_) and intention to stay (with *R^2^* = 0.466) than for autonomy support (with *R^2^* = 0.353 at T_2_ and *R^2^* = 0.302 at T_3_). The autoregressive standardized path coefficients of the variable professional identification between individual measurement time points are stronger than autoregressive path coefficients of the variable autonomy support. Of particular interest, however, are the two significant cross-path coefficients in the model:

- Professional identification at time T_1_ positively predicts perceived autonomy support at time T_2_ (*β* = 0.304, *p* < 0.01);
- Perceived autonomy support at time T_3_ positively predicts professional identification at time T_4_ (*β* = 0.160, *p* < 0.05).

Additionally, intention to stay at time point T_4_ was implemented into the model, being significantly predicted by professional identification at measurement time point T_2_ (*β* = 0.573, *p* < 0.001) and T_4_ (*β* = 0.679, *p* < 0.001). Yet for the latter it must be acknowledged that this relationship simply constitutes a correlation, hence indicated via a bidirectional path.

**Figure 3:**
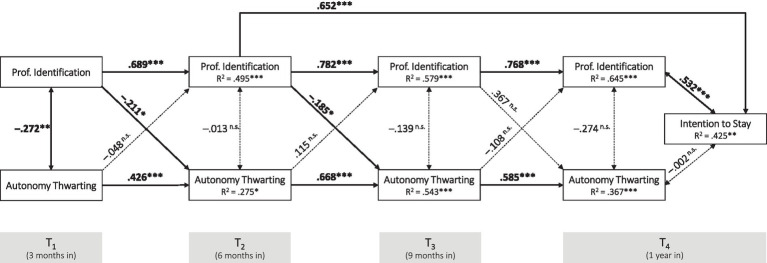
Cross-lagged panel model of professional identification and autonomy thwarting at four time points and intention to stay at T_4_ (Model 2). ***p ≤ 0.001, **p ≤ 0.01, *p ≤ 0.05.

Apart from the interrelationship of autonomy support with professional identification, we also analyzed the interrelationship of *professional identification* with *autonomy thwarting* over time and their relationship to the variable *intention to stay* at time T_4_. The corresponding cross-lagged panel model is presented in Figure 3. *Model 2* showed suboptimal model fit under the assumption of all possible paths, so the non-significant paths on variable *intention to stay* (T_4_) were removed. This model optimization resulted in a better model fit of the full model measured against the referenced information and goodness of fit criteria. Consequently, recommended cutoff values regarding the fit indexes for assessing model fit in structural equation modeling are met (Homburg and Baumgartner, 1995; Marsh et al., 2004; Homburg et al., 2008): *χ^2^* = 25.41, *df* = 17, *p* > 0.05 (with H_0_ = specified model, which therefore cannot be rejected); CFI = 0.960; TLI = 0.918; RMSEA = 0.079, RMSEA 90% CI: [0.00, 0.14]; SRMR = 0.085.

In this model as well, reasonable proportions of variances in the dependent variables were explained by the independent variables in the model. While the *coefficient of determination* increases over time with regard to professional identification (with *R^2^* = 0.495 at T_2_, *R^2^* = 0.579 at T_3_, and *R^2^* = 0.645 at T_4_), it is largest for the measurement time point T_3_ in terms of autonomy thwarting (with *R^2^* = 0.275 at T_2_, *R^2^* = 0.543 at T_3_, and *R^2^* = 0.367 at T_4_). The *R^2^* for intention to stay at measurement time point T_4_ equals 0.425.

Similar to *Model 1*, autoregressive path coefficients across the four measurement time points are (significantly) stronger for professional identification than for the autonomy-related variable, which in this case is autonomy thwarting. Again, we have two significant cross-path coefficients in this model, both indicating the same direction: professional identification at time T_1_ negatively predicts perceived autonomy thwarting at time T_2_ (*β* = −0.211, *p* < 0.05), and professional identification at time T_2_ negatively predicts perceived autonomy thwarting at time T_3_ (*β* = −0.185, *p* < 0.05). As expected from *Model 1*, again intention to stay at time point T_4_ is significantly predicted by professional identification at measurement time point T_2_ (*β* = 0.652, *p* < 0.001) and T_4_ (*β* = 0.532, *p* < 0.001). As outlined above, the latter relationship simply constitutes a correlation.

## 6. Discussion

### 6.1. Interpretation of findings in light of our hypotheses

In the following, we will discuss the results with respect to our hypotheses presented in Section 3. Testing the hypotheses by means of *Model 1* initially showed a medium^5^ and significant positive effect of professional identification at T_1_ (3 months in training) on perceived autonomy support at T_2_ (6 months in training). We therefore found evidence for our assumption of a *coherent effect* (*H4*). This effect, however, could not be demonstrated between measurement time points T_2_ and T_3_. Yet, we also found evidence for (*H1*) in *Model 1*, suggesting that greater perceived autonomy support through seminar teachers at T_3_ (9 months in training) lead to greater professional identification at T_4_. Despite its limited magnitude, the observed effect is still statistically meaningful. It seems that in the first third of teacher training, professional identification has an influence on the own perception of the experienced autonomy support (*H4*). This relationship changes, however, to the extent that trainee teachers’ perceived autonomy support positively predicts professional identification (*H1*) as soon as they enter the assessment phase and seminar teachers become reviewers, which may be due to the change in the seminar teacher’s role from advisor to assessor (see Sections 2.2.2 and 4.1). Salzmann et al. (2018) also found in their longitudinal study that perceived autonomy had a beneficial effect on the alteration of affective commitment towards the training profession, which included professional identification, during the latter half of the training duration. No supporting evidence could be found for a negative effect of perceived autonomy support through seminar teachers to professional identification among trainees (*H2*).

Hints for confirming *H4* can also be found in *Model 2*, as both, from measurement time point T_1_ to T_2_ as well as from measurement time point T_2_ to T_3_, perceived autonomy thwarting is negatively predicted by professional identification, each with a small but significant effect size. Consequently, a stronger identification with the teaching profession leads to a lower level of perceived autonomy thwarting in trainee teachers. From measurement time point T_3_ to T_4_ in *Model 2*, no significant cross-paths were found. Hence, *H3* must be rejected as a *higher* level of perceived autonomy thwarting through seminar teachers does not lead to lower professional identification among trainees. Regarding the presumed *contradictory effect* (*H5*), it can also be concluded that this hypothesis must be rejected. This is due to the lack of evidence indicating that a higher level of professional identification among trainees leads to a lower perception of autonomy support or a higher perception of autonomy thwarting.

According to our findings, a higher level of professional identification among trainees 6 months as well as 12 months in teacher training positively predicted their intention to remain in the teaching profession after 1 year in teacher training. This result supports *H6* and is consistent across both *Model 1* and *Model 2*. It is noteworthy that the relationship between professional identification and the intention to stay after 1 year is not statistically significant at both T_1_ (3 months in training) and T_3_ (9 months in training). *From a content-related perspective*, this could be attributed to the insufficient development of professional identification during the initial 3 months (T_1_), indicating that some trainees may not have fully embraced their new profession yet and therefore their professional identification might not align with their intention to stay at T_4_. However, after 6 months (T_2_), a different pattern emerges, indicating a significant predictive relationship. Another shift occurs 3 months later (T_3_), as trainee teachers enter a new phase of adjustment, which involves a lengthy summer break and transitioning into a role where they teach their own classes at the training school for the first time, assuming the responsibilities of a fully-fledged teacher (see Section 2.2.2). Consequently, it is possible that their professional identification observed at T_3_ may not necessarily correspond to the trainee teachers’ intention to remain in the profession at a later time point. Interestingly, professional identification appears to consolidate once again after a few months (T_4_), which is associated with a higher intention to stay at T_4_. *From a statistical perspective*, it seems plausible that the relatively strong influence of professional identification (T_2_ and T_4_) on intention to stay (T_4_) is responsible for the fact that professional identification (T_1_ and T_3_) on intention to stay (T_4_) no longer becomes significant in the overall model.^6^ This is especially true in light of the fact that the Pearson correlations between professional identification (T_1_ and T_3_) and intention to stay (T_4_) are weaker (see Table 1). Various studies confirm that the presence of a strong professional identification, which entails a profound sense of allegiance and dedication to the occupation, can decrease the likelihood of dropping out and enhance the likelihood of staying committed to it (e.g., van Dick and Wagner, 2002; Hong, 2010; Avanzi et al., 2014). However, it should be noted that our results do not show a significant relationship between intention to stay and either perceived autonomy support or perceived autonomy thwarting from seminar teachers. Therefore, *H7*, which suggests that higher perceived autonomy support or lower perceived autonomy thwarting through seminar teachers leads to a higher intention to stay in the profession after 1 year, is not supported by our data.

Lastly, we also want to refer to the autoregressive path coefficients of the two cross-lagged panel models: Clearly, these autoregressive path coefficients are stronger for professional identification than for autonomy support and autonomy thwarting. This means that the construct of professional identification is more stable, i.e., changes less over time, which is in line with the results of Maué et al. (2023), who also applied a cross-lagged panel analysis for, among other things, trainees’ identification in VET. This seems to be plausible since perceived autonomy support and thwarting are by definition highly dependent on context and situation (Patall et al., 2018; Vansteenkiste et al., 2020).

### 6.2. Limitations of the study

Although our longitudinal design allowed us to test relevant hypotheses related to the identified research desiderata and corresponding research questions, our study has some limitations that need to be stated and discussed. First, it should be noted that a full survey of all four VET teacher training locations in Baden-Württemberg was not possible for administrative reasons, leaving us with a relatively small sample in our study, for which representativeness is limited. This also prevented us from estimating our constructs in the models as latent factors. However, we surveyed a sample that is representative in sociodemographic data of the corresponding overall sample. Irrespective of the varying structures of teacher education across different federal states and countries, a profound process of professional identification unfolds during this phase (see Gracia et al., 2022). This fact underscores the relevance and applicability of our implications to other educational contexts as well. Second, while our quantitative design allows for statistical inference and hypothesis testing, it is not able to draw conclusions about the individually perceived quality of the relationship between participants (trainees) and the seminar teacher. Thus, it seems appropriate to back up our findings with perspective qualitative interview studies. Third, it should also be noted that in our study we considered subjectively perceived autonomy support or autonomy thwarting. On the one hand, it must be taken into account that perceived autonomy can sometimes differ from the autonomy actually granted. Yet, Meyer-Ahrens et al. (2010) found that the mere perception of autonomy may be more important than actual implementation. On the other hand, it should be kept in mind that we were only able to include the perspective of the trainee teachers, but not that of the seminar teachers in the data. Fourth, since trainees are typically supervised by two seminar teachers (one for each subject), assessments of perceived autonomy support or autonomy thwarting through the seminar teachers had to be assessed globally or rated on average by trainees. Particularly when there were significant differences in perception between the two seminar teachers, this could lead to distortions. However, using such a global assessment made the data collection phases more time-efficient and less demanding. Fifth, the participation rate decreased across the four survey time points, presumably because work-related time demands increased over time, leaving even less time to respond to our questionnaires. Hence, as outlined in Section 4.3, we implemented FIML as a method which can statistically compensate the missing data well. Sixth, three out of the four significant cross-lagged path beta coefficients (which represented *H1* to *H5*) were small in their effect size. Nonetheless, Orth et al. (2022, p. 1) recommend “to use 0.03 (small effect), 0.07 (medium effect), and 0.12 (large effect) as benchmark values when interpreting the size of cross-lagged effects.” This means that the significant cross paths of our models are large in effect size, thus even more weight could be attributed to them, especially against the background of the rather small sample. Lastly, it is not possible to draw a definitive causal conclusion for the specific impact of professional identification measured at time point T_4_ on intention to stay at T_4_. However, as evidenced by the effect of professional identification at T_2_ on the intention to stay at T_4_, we found indications for a causal relationship.

### 6.3. Implications for teacher training

Our findings suggest that the cultivation of a professional identity amongst trainee teachers may serve as an effective means of bolstering their commitment to the teaching profession. This highlights the importance of teacher education programs in developing not only the necessary skills and knowledge, but also a differentiated sense of professional identity and purpose, and thus the potential effectiveness of processes aimed at fostering such identity development. Accordingly, research in the field of teachers’ professional identity across different countries suggests that enhancing initial training for teachers should be a focus of future endeavors. Recognizing the crucial nature of this process, efforts should be made to foster a strong and cohesive professional identity among prospective teachers. This, in turn, can contribute to lower rates of attrition among trainee teachers and lead to improved educational outcomes during this critical stage (Gracia et al., 2022). The relevance of research on the formation of teachers’ professional identity lies, among other things, in the importance of mentoring, i.e., its potential to aid teacher educators, seminar teachers and mentors in better understanding and conceptualizing the necessary support for beginning teachers (Volkmann and Anderson, 1998; Hobson, 2002; Beijaard et al., 2004; Izadinia, 2016). Trainee teachers are considerably influenced by important professional reference persons. These persons play a decisive role in the formation of professional identity and support the trainee teachers in identifying with the professional field, internalizing professional values and norms, and developing professional and pedagogical competencies. Their support, guidance, as well as participatory practices can help trainee teachers form their professional identity and sense of self to anchor them in their role as teachers (see also Klotz et al., 2014). Indeed, instances where trainees undergo a shift in their professional identification often occur within the context of social interactions with instructors during teaching practice periods. Trainee teachers begin to recognize teacher training as a valuable resource and an integral part of their personal life project (Dahl, 2020).

In this regard, it also seems appropriate and worthwhile, for example, to implement suitable trainings for seminar teachers on autonomy-promoting support and relationship building. This appears to be particularly important because the concept of autonomy in educational sciences is inherently based on a certain ambivalence, which must be taken into account in the context of content-related discussions as well as in the promotion of educational practice: In principle, all demands for self-determined participation and autonomy involve the fundamental question of whether participation and autonomy are addressed or postulated as an end or as a means of educational or social practice. The former would be about supporting the development of “real” autonomous judgment and action competence. The latter would instrumentalize the need for self-determination to make people do or, more to the point, want what they are externally supposed to want (Heid, 1989; Kärner et al., 2023). The findings of this study also highlight that “the self” and “the other” should not be considered and perceived as independent entities but rather as interdependent constructs that mutually shape and influence each other (Sembill and Kärner, 2018). Hence, it is crucial to consider an intricate system of social interdependencies when analyzing the relational dynamics among trainee teachers and seminar teachers (Kärner et al., 2021b). To put this in more concrete terms—both trainee teachers and seminar teachers have needs and ideas about, for example, autonomy, social integration and the experience of competence, which need to be taken into account and, if necessary, balanced in the context of the respective social practice—and to take this one step further by following Heid (1999): It should be recognized that in organized educational work there are two independent persons acting: the teacher or educator on one side and the learner or person being educated on the other. The needs of the learner are not guidelines, but prerequisites that the teacher must meet in order to achieve his or her purpose, which is to ensure successful teaching. From the learner’s point of view, the teacher’s actions and requirements are nothing more than conditions that must be met in order to accomplish his or her goal of successful learning.

Finally, and particularly against the background of the shortage of (VET) teachers in Germany (Stellmacher et al., 2020) and across different countries (e.g., Keller et al., 2021; Meijer, 2021; Mičiulienė and Kovalčikienė, 2023), the promotion of professional identification processes in the sense of a teacher identity can be assessed as crucial. In this respect, an autonomy-supporting environment, e.g., created by seminar teachers, can already contribute to future retention rates during teacher training.

## Data availability statement

The datasets presented in this article are not readily available because the study was approved by the responsible governmental authority and disclosure of data is not permitted. Requests to access the datasets should be directed to JKW, ju.weiss@uni-hohenheim.de.

## Ethics statement

Ethical review and approval was not required for the study on human participants in accordance with the local legislation and institutional requirements. The patients/participants provided their written informed consent to participate in this study.

## Author contributions

JKW: conceptualization, methodology, software, formal analysis, data curation, writing—original draft, editing, visualization, writing—review and project administration. MB: conceptualization, methodology, software, formal analysis, data curation, writing—original draft, editing, visualization and writing—review. TK: writing—original draft, editing, and validation. All authors contributed to the article and approved the submitted version.

## Conflict of interest

The authors declare that the research was conducted in the absence of any commercial or financial relationships that could be construed as a potential conflict of interest.

## Publisher’s note

All claims expressed in this article are solely those of the authors and do not necessarily represent those of their affiliated organizations, or those of the publisher, the editors and the reviewers. Any product that may be evaluated in this article, or claim that may be made by its manufacturer, is not guaranteed or endorsed by the publisher.

## Footnotes

^1^Teacher education in Germany comprises two phases: academic education (e.g., at university) and *practical in-service training*. In the latter, trainee teachers are trained over 1–2 years (depending on the federal state) at a *training school* as well as at *college of didactics and teacher education (= seminar)*. Here, seminar teachers are responsible for training in didactics and teaching competence in specific subjects. They assume a dual role as advisor and assessor, which can lead to inter-role conflicts; see also Section 2.2.2. For a more detailed and summarizing description of the organizational context of VET teacher training in Germany see also Kärner et al. (2021a) or Kärner et al. (2021b).

^2^In this context, T*_n_* represents the measurement time point, of which there are four in total (T_1_, T_2_, T_3_, T_4_) with *n* = {1,2,3}.

^3^The analyses in this paper are based on a study in which a total of 86 trainee teachers from one training institution are voluntarily surveyed over the entire duration of teacher training. Since data on the variables of interest in this paper are not available from all participants, the sample is reduced.

^4^Regarding effect sizes we refer to Cohen (1988).

^5^Standardized beta coefficients can be interpreted like correlation coefficients (Peterson and Brown, 2005), which is why we also refer to Cohen (1988).

^6^If we model the cross-lagged model for only one of the paths from professional identification to intention to stay at a time, and disregard each of the other time points in relation to intention to stay, we indeed find that professional identification significantly positively predicts intention to stay at all four time points (T_1_: *β* = 0.318, *p* < 0.05; T_2_: *β* = 0.641, *p* < 0.001; T_3_: *β* = 0.446, *p* < 0.01; T_4_: *β* = 0.700, *p* < 0.001).

## Abbreviations

AS: Autonomy support
AT: Autonomy thwarting
BPNT: Basic psychological need theory
BW: Baden-Württemberg
FIML: Full information maximum likelihood method
Prof. Identification: Professional identification
SCT: Self-categorization theory
SDT: Self-determination theory
SIT: Social identity theory
T: Measurement time point
VET: Vocational education and training
